# Analysis of Agreement on Traditional Chinese Medical Diagnostics for Many Practitioners

**DOI:** 10.1155/2012/178081

**Published:** 2011-06-30

**Authors:** Lun-Chien Lo, Tsung-Lin Cheng, You-Chieh Huang, Ying-Ling Chen, Jeng-Ting Wang

**Affiliations:** ^1^Department of TCM, Changhua Christian Hospital, 135 Nanxiao Street, Changhua City 500, Taiwan; ^2^Graduate Institute of Statistics and Information Science, National Changhua University of Education, No. 1, Jin-De Road, Changhua City 500, Taiwan

## Abstract

In Traditional Chinese Medicine (TCM) diagnostics, it is an important issue to study the degree of agreement among several distinct practitioners. In order to study the reliability of TCM diagnostics, we have to design an experiment to simultaneously deal with both of the cases when the data is ordinal and when there are many TCM practitioners. In this study, we consider a reliability measure called “Krippendorff's alpha” to investigate the agreement of tongue diagnostics in TCM. Besides, since it is not easy to obtain a large data set with patients rated simultaneously by many TCM practitioners, we use the renowned “bootstrapping” to obtain a 95% confidence interval for the Krippendorff's alpha. The estimated Krippendorff's alpha for the agreement among ten physicians that discerned fifteen randomly chosen patients is 0.7343, and the 95% bootstrapping confidence interval for the true alpha coefficient is [0.6570, 0.7349]. The data was collected and analyzed at the Department of Traditional Chinese Medicine, Changhua Christian Hospital (CCH) in Taiwan.

## 1. Introduction

Studying reliability and validity is important in designing questionnaires in psychological research. The practitioners of western medical system are often skeptical about objectivity of clinical examination in TCM. In TCM diagnostics, there are four clinical diagnostics to evaluate a patient's health condition, which include “Inspection,” “Smelling and Listening,” “Inquiring,” and “Palpation.” The outcome of tongue inspection is an index among many important characteristics in TCM diagnostics. In general, the tongue inspection in TCM refers to the shape, luxuriance and witheredness, toughness and softness, thinness and swelling, and so forth. For example, a patient having an enlarged tongue with slippery fur is categorized into the Yang deficiency and requires corresponding TCM treatment. The diagnostic of TCM depends mainly on the sensorial evaluation. Therefore, the reliability and objectivity of such sensorial diagnostics is important in the modernization of the TCM theory since unreliable diagnoses lead to inappropriate prescriptions. 

 To compare with western modern medical research, only few attempts have so far been made at agreement analysis in TCM diagnostics. In Kim et al. [1], the authors examine the reliability of TCM tongue inspection by the evaluation of inter- and intrapractitioner agreement levels for specific tongue characteristics. Mist et al. [2] investigates whether a training process that focused on a questionnaire-based diagnosis in TCM would improve the agreement of TCM diagnoses. Zhang et al. [3] studied the effect of training that aims to improve the agreement in TCM diagnosis among practitioners for persons with the conventional diagnosis of rheumatoid arthritis. The above studies used proportion of agreement, similar to Goodman and Kruskal [4], to express the degree of agreement among the TCM practitioners. While the proportion of agreement is widely used, such a statistic overlooks the possibility that randomness might cause agreement and/or disagreement. This problem has been partly solved by Cohen [5] who invented the renowned “kappa” coefficient to measure agreement between two raters. Since Cohen's kappa deals only with binary or nominal data, it does not take the discrepancy of agreement for different categories into account. The degree of disagreement may vary according to the categories that classify the data. For example, if patients' health condition can be categorized into “very good,” “ordinary,” and “severely bad,” the agreement between a rating of “very good” and a rating of “ordinary” differs from the agreement between a rating of “very good” and a rating of “severely bad.” O'Brien et al. [6] studied the reliability of diagnostic variables in a TCM examination. In their study, they used the Cohen's kappa to measure the agreement among three TCM practitioners and suggest that even when there are certain features of the TCM system that are highly objective and repeatable, there are also other features that are subjective and unreliable. However, Cohen's kappa cannot deal with the ordinal data. Weighted kappa [7] is a generalization of the original kappa, and it uses the same contingent table to describe the data. However, the weighted kappa cannot deal with the cases when there are more than two raters. Fleiss [8] proposed another “kappa” to measure agreement among more than two practitioners while it only works for nominal data. In the study of the reliability of TCM diagnostics which discerns ordinal categories, not only the levels of disagreements but also the generalization to the case of more than two practitioners should be taken into account simultaneously. To overcome both difficulties, Krippendorff's alpha [9–12] emerges as a good substitute for both of the Cohen's kappa and Fleiss kappa. 

In this study, we recruited 10 TCM physicians with ages ranging from 28 to 46 and randomly chose 15 patients taking TCM treatments in CCH. Each patient's tongue is photographed using digital camera. Then the recruited TCM practitioners independently classified the patient's tongues into three categories: thin tongue, normal tongue, and enlarged tongue. The estimated Krippendorff's alpha is 0.7343 and its 95% confidence interval by a modified bootstrapping is [0.6570, 0.7349]. We will report the results in next section. 

## 2. Method

### 2.1. Patients and TCM Tongue Inspectors

Fifteen patients were recruited randomly from the archive of the Department of Traditional Chinese Medicine (TCM), Changhua Christian Hospital (CCH). Their tongues were photographed by a digital camera and were rated, within a day, by ten TCM practitioners educated in China Medical University, Taiwan. All of the recruited TCM practitioners have passed the National Professional & Technical Examinations for Doctors of Chinese Medicine. The rating levels are classified into three categories: enlarged tongue, normal (moderate) tongue, and thin tongue. In general, an enlarged tongue and a thin tongue indicate unhealthy conditions. The ages of the TCM practitioners range from 30 to 45. About five of them just graduated from the medical school within 5 years, and the other five are senior TCM physicians in Changhua Christian Hospital. 

### 2.2. Statistical Analysis

Cohen's kappa is a popular measure of agreement, and its confidence interval relies on a large sample which is, in general, hard to obtain in medical study. Cohen [5] proposed an algorithm based on bootstrapping to obtain a 95% confidence interval for Krippendorff's alpha. In our setting, the algorithm cannot be directly applied and requires some modification such that it can comprise the estimated Krippendorff's alpha. A concrete example on how to calculate Krippendorff's alpha can be found in Cohen [5, 13]. The Krippendorff's alpha measure for tongue inspection data obtained in the Department of Chinese Medicine in Changhua Christian Hospital of Taiwan, using nominal weight, is about 0.7343. 

In general, people applied asymptotic normality to obtain confidence interval when the data at hand is large enough. While in medical study, it is not easy to obtain a large sample with many raters and many patients in a clinical trial. When we are confronted with a small sample, we may apply Efron's bootstrapping [14] to obtain a reasonable confidence interval for Krippendorff's alpha that measures the agreement of diagnostics among raters. On modifying Krippendorff's original algorithm, we may obtain a reasonable 95% confidence interval for the true Krippendorff's alpha (Appendix B). 

**Figure 1:**
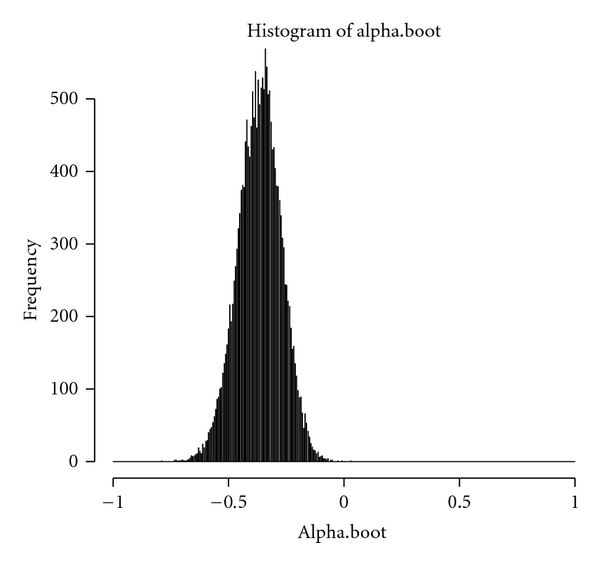
The distribution of bootstrapped *α* adopting Krippendorff's original algorithm.

**Figure 2:**
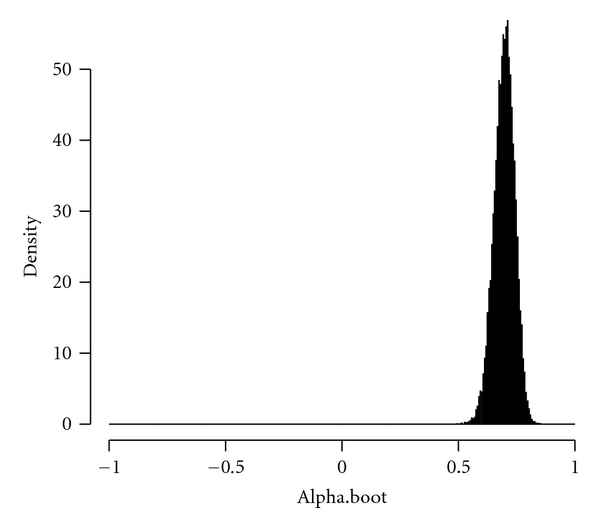
The distribution of bootstrapped *α* adopting our modified algorithm.

Table 1 is the data of tongue inspection obtained in the Department of Chinese Medicine, Changhua Christian Hospital of Taiwan.  Figure 1 reports the 95% confidence interval for Krippendorff's alpha for the tongue inspection data by Krippendorff's original algorithm. From Figure 1, we see that the confidence interval using Krippendorff's original algorithm does not include the estimated Krippendorff's alpha = 0.7343. However, from Figure 2, the 95% confidence interval of bootstrapped *α* using our modified algorithm contains the estimated Krippendorff's alpha. 

## 3. Conclusion

There are many works investigating agreement measures for western medical diagnostics, while only few study agreement analysis among TCM physicians. In the literature concerning agreement analysis, although many researchers consider complex TCM diagnostics, most of them adopted a so-called “proportion of agreement” measure which overlooks the possible bias caused by randomness. O'Brien et al. [6] used the Cohen's kappa to measure the agreement among three TCM practitioners while Cohen's kappa cannot deal with data of ordinal scale. To simultaneously deal with the case when there are many raters and the case when the data is ordinal as well as multinomial distributed, Krippendorff's alpha provides itself as a good substitute both for Cohen's and Fleiss' kappa. We not only estimate the Krippendorff's alpha coefficient of 0.7343 for the tongue inspection data obtained in the Department of TCM, CCH of Taiwan, but also modify Krippendorff's bootstrapping algorithm to obtain a 95% confidence interval [0.6570, 0.7349] for the Krippendorff's alpha. In this study, for such a dataset that a patient's tongue is classified into three distinct categories, it seems that the diagnostics of tongue's shapes in TCM is moderately reliable in the standard of reliability requirement. Apart from tongue inspection, there are many other diagnostics that are regularly used to rate a patient's health condition, for example, listening, smelling, inquiring, palpation, and so forth. The agreement analysis of other diagnostics in TCM among many practitioners involves more complicated methods of experimental design. This study may serve itself as a touchstone of approaching the reliabilities of many other diagnostics among several practitioners in TCM. We will focus on this topic in the future.

## Figures and Tables

**Table 1:** Tongue diagnostics obtained by Changhua Christian Hospital.

Unit	1	2	3	4	5	6	7	8	9	10	11	12	13	14	15
Rater 1	2	3	1	1	3	3	1	1	1	2	3	1	2	2	3
Rater 2	2	3	1	2	3	3	1	1	1	2	3	1	2	2	3
Rater 3	2	3	1	2	3	3	1	1	1	2	3	1	2	2	3
Rater 4	2	3	1	2	3	3	1	1	2	2	3	1	2	2	3
Rater 5	2	3	1	2	3	3	1	2	2	2	3	1	2	2	3
Rater 6	2	3	1	2	3	3	1	2	2	2	3	2	2	2	3
Rater 7	2	3	1	2	3	3	1	2	2	2	3	2	3	2	3
Rater 8	2	3	1	2	3	3	2	2	2	2	3	2	3	2	3
Rater 9	2	3	1	2	3	3	2	2	3	2	2	2	3	3	3
Rater 10	2	3	2	3	3	3	2	2	3	3	2	2	3	3	2

**Table:** (a)

Rater	Unit			
	1	2	⋯	*N*
1	*c*_11_	*c*_12_	⋯	*c*_1*r*_
2	*c*_21_	*c*_22_	⋯	*c*_2*r*_
⋮	⋮	⋮	⋱	⋮
*r*	*c*_*r*1_	*c*_*r*2_	⋯	*c*_*rN*_

Number of ratings	*m*_1_	*m*_2_	⋯	*m*_*N*_

**Table:** (b)

	1	⋯	*j*	·
1	*o*_11_	·	*o*_1*j*_	⋯
·	·		·	⋮
*i*	*o*_*i*1_	·	*o*_*ij*_	⋯
·	·		·	⋱

## Appendices

### A.

See Table 1 and Figures 1 and 2.

### B. A Modified Algorithm for Bootstrapping Krippendorff's Alpha

To calculate Krippendorff's alpha, firstly, the observations must be arranged and recorded in the form of Table 2(a). In this table, suppose that there are *k* categories under consideration, and *c*_*ij*_ stands for the category that rater *i* attributes to unit *j*, and *m*_*u*_ is the number of raters that categorizes unit *u*. Secondly, we tabulate coincidences within units by Table 2(a).

Let *P*_*n*_^*m*^ = *m*(*m* − 1)⋯(*m* − *n* + 1). The number *o*_*ij*_, 1 ≤ *i*, *j* ≤ *k*, is defined by

(B.1) $$o_{ij}=\{\begin{array}{cc} \underset{u}{\sum }m_{u}\frac{P_{2}^{m_{i}}}{P_{2}^{m_{u}}}, & i=j,\\ \underset{u}{\sum }m_{u}\frac{P_{1}^{m_{i}}P_{1}^{m_{j}}}{P_{2}^{m_{u}}}, & i\neq j. \end{array}$$

On the other hand, we define the marginal *s*_*i*_ = ∑_*j*_ *o*_*ij*_ and *s* = ∑_*i*_ *s*_*i*_ = ∑_*i*_∑_*j*_ *o*_*ij*_ is the sum of the marginal *n*_*i*_. From the table of coincidence and by the definition of *o*_*ij*_, we may obtain the observed disagreement measure *D*_*o*_. In fact, *o*_*ij*_ represents the number of pairs (*i*, *j*) that is rated by the raters, and *s*_*i*_ stands for the number of units that are classified into the *i*th category. In this step, we also have to define the expected disagreement measure  *D*_*e*_. The notion of expected disagreement measure can be understood via drawing balls from urns. Suppose that there are *s* balls in an urn. Among the *s* balls, there are *s*_*j*_ balls that are numbered *j* = 1,2,…, *k* and ∑_*j*_ *s*_*j*_ = *s*. The expected agreement matrix consists of the entries *e*_*ij*_ which are formed by

(B.2) $$e_{ij}=\{\begin{array}{cc} s\frac{P_{2}^{s_{i}}}{P_{2}^{s}}, & i=j,\\ s\frac{P_{1}^{s_{i}}P_{1}^{s_{j}}}{P_{2}^{s}}, & i\neq j. \end{array}$$

Thirdly, we define the ordinal metric differences which are weights put on the differences between ranks. In general, we know that obtaining Grade A is different from obtaining Grade B. Moreover, the difference between Grade A and Grade B is smaller than that between Grade A and Grade C. Therefore, disagreement measure should depend on the difference of categories. In Krippendorff [5, 13], the author suggests many metric differences, for example, interval metric differences, ratio metric differences, circular metric differences, and bipolar metric differences. In this paper, for clarity and convenience, we adopt the weights by interval metric differences which are defined by

(B.3) δij2interval=(i−j)2.

Finally, the Krippendorff's alpha is then defined by

(B.4) α=1−DoDe=1−∑i∑joijδij2interval∑i∑jeijδij2interval.

The following algorithm is a modified bootstrapping method for obtaining a 95% confidence interval for the Krippendorff's alpha.

Step 1 Deriving the concordance matrix, observed disagreement statistics *D*_*o*_, expected disagreement measure *D*_*e*_, and weight _interval_ *δ*_*ij*_^2^ = (*i* − *j*)^2^.

Step 2 Define _metric_ *δ*_*ck*_^2^ = *F*(*R*), where *R* is randomly drawn from [0,1] within a continuum with a finite precision. That continuum is segmented by the probabilities  *p*_*ck*_ = *o*_*ck*_/*n* so that each *R* in segment *p*_*ck*_ is associated with the corresponding _interval_ *δ*_*ck*_^2^.

Step 3 Let the number *M* of draws be (1/2)∑_*u*_ *m*_*u*_(1 − *m*_*u*_). Then consider the number of possible observed disagreement frequency to be $\tilde{M}=(D_{o}s/M$) in every replicate.

Step 4 Bootstrap the distribution of *α* as follows.

Set the array *N*_*α*_ = 0, where − 1 ≤ *α* ≤ 1, and *α* has at least 4 significant digits.
Do *X* replicates, in which default *X* = 20,000.
Set SUM = 0.
Do *M* times.
  Pick a random number between 0 and 1 (uniform distribution).
  Determine _interval_ *δ*_*ck*_^2^ by means of the function *F*(*R*).
  SUM ≤ SUM + _interval_ *δ*_*ck*_^2^.
  *α* = 1 − ((SUM · *n*)/*D*_*e*_ *M*).
  If *α* < −1.000, *N*_*α*=−1_ ≤ *N*_*α*=−1_ + 1.
Otherwise, *N*_*α*_ ≤ *N*_*α*_ + 1.
